# A Comparison of Error Bounds for a Nonlinear Tracking System with Detection Probability P_d_ < 1

**DOI:** 10.3390/s121217390

**Published:** 2012-12-14

**Authors:** Huisi Tong, Hao Zhang, Huadong Meng, Xiqin Wang

**Affiliations:** Department of Electronic Engineering, Tsinghua University, Beijing 100084, China; E-Mails: haozhang@tsinghua.edu.cn (H.Z.); menghd@tsinghua.edu.cn (H.M.); wangxq_ee@tsinghua.edu.cn (X.W.)

**Keywords:** posterior Cramer-Rao lower bound (PCRLB), detection probability, target state estimation, random finite set (RFS), information reduction factor (IRF)

## Abstract

Error bounds for nonlinear filtering are very important for performance evaluation and sensor management. This paper presents a comparative study of three error bounds for tracking filtering, when the detection probability is less than unity. One of these bounds is the random finite set (RFS) bound, which is deduced within the framework of finite set statistics. The others, which are the information reduction factor (IRF) posterior Cramer-Rao lower bound (PCRLB) and enumeration method (ENUM) PCRLB are introduced within the framework of finite vector statistics. In this paper, we deduce two propositions and prove that the RFS bound is equal to the ENUM PCRLB, while it is tighter than the IRF PCRLB, when the target exists from the beginning to the end. Considering the disappearance of existing targets and the appearance of new targets, the RFS bound is tighter than both IRF PCRLB and ENUM PCRLB with time, by introducing the uncertainty of target existence. The theory is illustrated by two nonlinear tracking applications: ballistic object tracking and bearings-only tracking. The simulation studies confirm the theory and reveal the relationship among the three bounds.

## 1. Introduction

In the Bayesian framework, the complete posterior density of the state is necessary, in order to obtain the optimal recursive random state estimate for a classical nonlinear filtering problem by using various sensors [1], but this problem has no analytic closed-form solution. Therefore, in practical applications nonlinear filtering by some form of approximation is performed, such as sequential Monte Carlo estimation [2]. Although a closed-form solution is absent, the best achievable second-order error performance for nonlinear filtering can be limited by an effective error bound [3].

Error bounds for nonlinear filtering can be applied in many fields. Firstly, error bounds can be used as a performance evaluation of suboptimal nonlinear filters and as a judgment of the effects of introduced approximations. For example, error bounds were applied in the cases of bearings-only tracking by a moving platform carrying sensor [4] and ballistic target tracking [5]. Secondly, an error bound was also applied as a tool in sensor system design [6], as it provides a guide to best achievable performance and help in sensors management.

There is a long history of the development of the error bounds for nonlinear filtering. Initially, the Cramér-Rao lower bound (CRLB) was introduced as a bound of the estimation when the state dynamics are deterministic, and a comprehensive review of pre-1989 attempts are presented in [7]. In 1998, [8] was considered as a key development for recursive calculation of the Fisher information matrix (the inverse of the CRLB) with further extensions applicable to a larger class of nonlinear models given in [9]. Because the state dynamics are modeled as being a stochastic vector with process noise, the bounds of [8] and [9] are referred to as Posterior Cramér-Rao lower bound (PCRLB). The PCRLB in [8] and [9] relies on the assumption that the probability of detection *P_D_* = 1 and the false alarm probability *P_FA_* = 0.

The assumptions of the sensors where *P_D_* = 1 and *P_FA_* = 0 are unrealistic in target tracking applications. The realistic scenarios are the cases where *P_D_* < 1 and *P_FA_* > 0, but this introduces an additional level of complexity for any estimation method, because it is necessary to consider the effect of the uncertainty in the sensor measurement origin, in addition to the uncertainty in the random target state. In [10], the effect of uncertain measurements is to multiply the Fisher information matrix by a constant factor less than unity, and this factor is referred to as the information reduction factor (IRF). Another PCRLB has been introduced in [11] for nonlinear filtering in the case where *P_D_* < 1 and *P_FA_* = 0. The solution is based on the enumeration of all possible miss/detection sequences. The bound has been proved as the exact bound via numerical simulations [12]. The method to calculate this bound is named Enumeration Method (ENUM). ENUM PCRLB is tighter than IRF PCRLB, but the computational complexity of ENUM PCRLB grows exponentially with time. Therefore, as discussed in [12], ENUM PCRLB is computationally feasible for a small prediction time, while IRF PCRLB is more suitable to an extended prediction time.

All the bounds mentioned above are based on the assumption that the target exists from the beginning to the end. However, the appearance of the target varies with time in many practical situations. Moreover, we cannot determine whether the target exists or not from the measurements, because it is unlikely to know whether there is missing detection or there are false measurements, especially in defense and surveillance [13,14]. Traditionally, data association is introduced to solve this problem and apply the PCRLB, but it is difficult to ensure that data association is right. Recently, to extend the single-target Bayesian formula to multiple targets, target states and sensor measurements are modeled as random finite sets (RFS) [15]. There are many tracking algorithms, such as [16] and [17], based on RFS statistics, and they generally have ignored the issue of data association.

Because both target states and estimates are modeled as RFS, traditional Euclidean distance could not be applied for calculating the error. Therefore, a meaningful distance named Optimal Sub-pattern Assignment (OSPA) distance in [18] and [19] is defined. This definition has been widely used in tracking algorithms and their performance evaluation ([16] and [20]).

For the problem of the error bound in the framework of finite set statistics, [18] has given a non-recursive bound. In our work [20], we use random set models and OSPA to deduce a recursive error bound for a tracking system, and the bound is named RFS bound. When the RFS bound is deduced, the disappearance of existing targets and the appearance of new targets are taken into account. This problem is very important in defense and surveillance [13], since the uncertainty of targets has a great impact on the calculation of error bounds.

The paper presents a comparative study of the RFS bound in [20] and the PCRLBs in the case where detection probability *P_D_* < 1, such as IRF PCRLB and ENUM PCRLB. We discuss this problem in two cases, one is when the target exists from the beginning to the end, and the other is when new targets might appear and existing targets could disappear. For the first case, we deduce two propositions. They prove that the RFS bound is equal to the ENUM PCRLB with four conditions and is always tighter than the IRF PCRLB. For the second model, these three bounds are hard to compare directly both quantitatively and qualitatively. Fortunately, their relationship is illustrated by two target tracking applications: ballistic object tracking and bearings-only tracking. Finally, these theoretical results are confirmed by simulations. Moreover, these examples reveal that the RFS bounds are tighter than the IRF PCRLB and ENUM PCRLB as the scan number increases, by introducing the uncertainty of target existence.

It is noted that the result in this paper is for the condition of sensors where *P_D_* < 1 and *P_FA_* = 0. The detection event given by a false alarm is omitted because the probability of false alarm is much smaller than the detection probability, such as in a typical radar system *P_d_* = 0.9 and *P_FA_* = 10^−6^, as indicated in [11] and [12]. The case where *P_D_* < 1 and *P_FA_* > 0 will be examined in future work.

In this paper, Section 2 introduces some background knowledge about the dynamic and sensor models, the PCRLB and the main theoretical results of ENUM PRRLB and IRF PCRLB. Section 3 reviews the basic knowledge of random set statistics, the random set dynamic and measurement models and the RFS bound. Section 4 compares these three bounds in two cases: when the target exists from the beginning to the end; and when targets might appear or disappear. Section 5 is devoted to the application examples: the tracking of ballistic missiles in the re-entry phase and bearings only tracking. Conclusions are given in Section 6.

## 2. The Bounds for Random Vector Estimation with P_D_ < 1

### 2.1. State and Measurement Radom Vector Models

For a discrete-time nonlinear filtering problem, the target state is modeled as a random vector, and the state dynamic equation is given by:

(1) $$x_{k+1}=f_{k}\left(x_{k}\right)+w_{k}$$

where **x***_k_* ∈ *R^m^* is the target state at time step *k*, *m* is the dimensionality of the target state, **f***_k_* is the state transition function, and **w***_k_* is a zero-mean white Gaussian process noise, with covariance matrix **Q***_k_*.

The sensor measurement model is a function of the target state, in which there is a single sensor and a single measurement at each time step. There are no false alarms *P_FA_* = 0, while there can be missed detections *P_D_* < 1. Each target only generates one measurement, and when the target is detected, the measurement equation is given by:

(2) $$z_{k}=h_{k}\left(x_{k}\right)+v_{k}$$

where **z***_k_* ∈ *R^r^* is the observation at time step *k*, **h***_k_* is the non-linear observation function, and **v***_k_* is a zero-mean white Gaussian noise, with covariance matrix **R***_k_*.

### 2.2. The PCRLB

The covariance of the estimate of **x***_k_* is **x̂***_k_* is an unbiased state estimator based on the sequence of sensor measurements {**z**_1_,⋯,**z***_k_*} before time step *k*. This estimator has a lower bound expressed as follows [21]:

(3) $$C_{k}=E\left[\left({\hat{x}}_{k}-x_{k}\right){\left({\hat{x}}_{k}-x_{k}\right)}^{T}\right]\geq J_{k}^{-1}$$

where **J***_k_* is referred to as the Fisher information matrix (FIM), and the **P***_k_* = **J***_k_*^−1^ is the PCRLB. The inequality in (3) means that the difference **C***_k_* − **J***_k_*^−1^ is a positive semi-definite matrix.

As in [11], for the state dynamics equation in (1) and the measurement equation in (2), the recursive formula of FIM is as follows:

(4) $$J_{k+1}=D_{k}^{33}-D_{k}^{21}{\left(J_{k}+D_{k}^{11}\right)}^{-1}D_{k}^{12}+J_{Z}\left(k+1\right)$$

where:

(5) $$D_{k}^{11}=E\left\{F_{k}^{T}Q_{k}^{-1}F_{k}\right\}$$

(6) $$D_{k}^{12}=-E\left\{F_{k}^{T}\right\}Q_{k}^{-1}={\left[D_{k}^{12}\right]}^{T}$$

(7) $$D_{k}^{33}=Q_{k}^{-1}$$

(8) $$J_{Z}\left(k+1\right)=E\left\{H_{k+1}^{T}R_{k+1}^{-1}H_{k+1}\right\}$$

The matrices **F***_k_* and **H***_k_* are respectively the Jacobians [8] of nonlinear functions **f***_k_* and **h***_k_*, which are defined as:

(9) $$\begin{array}{l} F_{k}={\left[\nabla_{x_{k}}{\left[f_{k}\left(x_{k}\right)\right]}^{T}\right]}^{T}\\ H_{k}={\left[\nabla_{x_{k}}{\left[h_{k}\left(x_{k}\right)\right]}^{T}\right]}^{T} \end{array}$$

As defined in [8], ∇ and Δ are operators of the first and second-order partial derivatives, respectively:

(10) $$\nabla_{x}={\left[\frac{\partial }{\partial x_{1}},\cdots ,\frac{\partial }{\partial x_{m}}\right]}^{T}$$

(11) $$\Delta_{x}^{x}=\nabla_{x}\nabla_{x}^{T}$$

where **x** is an m-dimensional estimated random parameter.

The initial FIM is calculated from the prior probability function *p*_0_(**x**_0_):

(12) $$J_{0}=E\left\{-\Delta_{x_{0}}^{x_{0}}\text{log}p_{0}\left(x_{0}\right)\right\}$$

### 2.3. Information Reduction Factor PCRLB

For the case where *P_D_* < 1, as in [10], the effect of potential missed detections is to scale the measurement error covariance by a factor 1/*P_D_*. In the terminology of [10], this scale factor is referred to as an “information reduction factor (IRF).” Hence, the recursive FIM is given by:

(13) $$J_{k+1}=Q_{k}^{-1}-Q_{k}^{-1}E\left\{F_{k}\right\}{\left[J_{k}+E\left\{F_{k}^{T}Q_{k}^{-1}F_{k}\right\}\right]}^{-1}E\left\{F_{k}^{T}\right\}Q_{k}^{-1}+P_{D}E\left\{H_{k+1}^{T}R_{k+1}^{-1}H_{k+1}\right\}$$

Then, the PCRLB calculated by IRF method is denoted by **P***_k_* (*IRF*), and is given by **P***_k_* (*IRF*) = **J***_k_*^−1^.

### 2.4. Enumeration Method PCRLB

In [11], the authors worked on the problem of calculating the PCRLBs for the cases where *P_D_* < 1 and *P_FA_* = 0. They introduced the detection/miss-detection sequences to determine the PCRLB. Here *S_i_* is a detection/miss-detection sequence.

The uncertain target dynamics is as:

(14) $$J_{k+1}\left(S_{i}\right)=Q_{k}^{-1}-Q_{k}^{-1}E\left\{F_{k}\right\}{\left[J_{k}+E\left\{F_{k}^{T}Q_{k}^{-1}F_{k}\right\}\right]}^{-1}E\left\{F_{k}^{T}\right\}Q_{k}^{-1}+d_{k+1}\left(S_{i}\right)E\left\{H_{k+1}^{T}R_{k+1}^{-1}H_{k+1}\right\}$$

where *d_k_*_+ 1_(*S_i_*) is defined in:

(15) $$d_{k}\left(S_{i}\right)=\{\begin{array}{l} 1,\;\text{if}\;\text{there}\;\text{is}\;\text{a}\;\text{detection}\;\text{at}\;\text{time}\;\text{k}\;\text{in}\;\text{the}\;\text{sequence}\;S_{i}\\ 0,\text{otherwise} \end{array}$$

Then, as introduced in [11], the PCRLB calculated by Enumeration method is denoted as **P***_k_*(*ENUM*), and is given by:

(16) $$P_{k}\left(\mathrm{ENUM}\right)=E_{S_{i}}\left[J_{k}{\left(S_{i}\right)}^{-1}\right]$$

At time step k, there are 2*^k^* possible scenarios *S_i_*. Therefore, **P***_k_*(*ENUM*) is the average over the scenario dependent bounds **J***_k_*(*S_i_*)^−1^.

## 3. The Bound for Random Set Estimation with P_d_ < 1

### 3.1. Random Finite Set

Random finite set (RFS) is a random variable which takes value as a finite set [18]. The element of this set is an unordered random variable and the number of elements is random and finite. Finite set statistics (FISST) were developed by Mahler and are widely considered as an effective tool for multi-target tracking systems. In the perspective of modeling the tracking system, two types of RFS are often used: Poisson RFS and Bernoulli RFS. Based on the model of Poisson RFS, a filter named Probability Hypothesis Density (PHD) filter [15] is applied in several fields [16,17]. However, PHD is a first-order statistical moment of the multi-target posterior probability, but not the posterior probability itself.

The filter derived from Bernoulli RFS attracts substantial interest and is used widely recently [18,20]. As in [18], here a Bernoulli RFS on a space *S* is defined by two parameters *b* and *ψ*, where 0 ≤ b ≤ 1 and *ψ*(•) is a density on *X*:

(17) $$f\left(X\right)=\{\begin{array}{cc} 1-b, & X=\emptyset ;\\ b\psi \left(x\right), & X=\left\{x\right\};\\ 0, & \mathrm{otherwise}. \end{array}$$

where the *f*(*X*) is the density of the RFS *X* in the space of finite sets.

For the function *g* taking value on the set *X*, the set integral of this function is:

(18) $$\int_{S}g\left(X\right)\delta X=g\left(\emptyset \right)+\sum_{n=1}^{\infty }\frac{1}{n!}\int_{S^{n}}g\left(\left\{x_{1},\cdots ,x_{n}\right\}\right)dx_{1},\cdots ,dx_{n}$$

The expectation of the function *h* on a RFS of density *g* is:

(19) $$E\left[h\right]=\int_{S}h\left(X\right)g\left(X\right)\delta X$$

For the error between the set *X* and its estimation *X̂*(*Z*), we should first define the distance between these two sets. This distance is based on the optimal sub pattern assignment (OSPA) metric [19]. For there is a single sensor and a maximum of a single measurement at each time step, the measurement set is *Z* and the OSPA error is defined as in [18]:

(20) $$\Sigma =E\left[e\left(X,\hat{X}\left(Z\right)\right)e{\left(X,\hat{X}\left(Z\right)\right)}^{T}\right]=\iint e\left(X,\hat{X}\left(Z\right)\right)e{\left(X,\hat{X}\left(Z\right)\right)}^{T}p\left(X,Z\right)\delta X\delta Z$$

where:

(21) $$e\left(X=\left\{x\right\},\;\;\;\;\hat{X}\left(Z\right)=\left\{\hat{x}\right\}\right)=x-\hat{x}$$

(22) $$e\left(X=\emptyset ,\;\;\;\;\hat{X}\left(Z\right)=\left\{{\hat{x}}^{\prime }\right\}\right)=e_{0},\;\;\mathrm{for}\;\mathrm{any}\;x^{\prime }$$

(23) $$e\left(X=\left\{x\right\},\;\;\;\;\;\;\hat{X}\left(Z\right)=\emptyset \right)=e_{1},\;\;\mathrm{for}\;\mathrm{any}\;x$$

(24) $$e\left(X=\emptyset ,\;\;\;\;\;\;\;\hat{X}\left(Z\right)=\emptyset \right)=0$$

For the cardinality of *X* and the estimation set *X̂*(*Z*) may be zero or one, (22) and (23) are defined to the error in cardinality mismatches.

### 3.2. State and Measurement Radom Set Models

The state dynamics and measurement model are as similar as what in [20]. Since the target in either in “present” or “absent” state, the state of the target is modeled by Bernoulli RFS as introduced in (17).

For the dynamical model, the Markov transition density is defined by:

(25) $$f\left(X_{k+1}|X_{k}=\left\{x_{k}\right\}\right)=\{\begin{array}{cc} r\psi \left(x_{k+1}|x_{k}\right), & X_{k+1}=\left\{x_{k+1}\right\}\\ 1-r, & X_{k+1}=\emptyset \end{array}$$

and:

(26) $$f\left(X_{k+1}|X_{k}=\emptyset \right)=\{\begin{array}{cc} \left(1-r\right)p_{0}\left(x_{k+1}\right), & X_{k+1}=\left\{x_{k+1}\right\}\\ r, & X_{k+1}=\emptyset \end{array}$$

where *r* ∈ [0,1] represents the probability of the state of the target at time step *k* + 1 surviving from the state at time-step *k* or remains empty. It means, conditional upon *X_k_* = {**x***_k_*}, that this target disappears with a probability of 1 − *r*. If there is no target at time-step *k*, a new target would bear with a probability of 1 − *r*. The target surviving probability is *r* means that the cardinality of target state set remains one form time *k* to *k* + 1 with a probability of *r*, while the probability of keeping no target is *r* means that the cardinality keeps zero form time *k* to *k* + 1 with a probability of *r*. Therefore, *r* is called maintenance probability of the cardinality of target state set. *ψ*(**x***_k_*|**x***_k_*_−1_) is the probability density of a transition from **x***_k_* state to **x***_k_*_+ 1_, which can be calculated by (1).

The prior probability function of the state set is also Bernoulli RFS:

(27) $$f\left(X_{0}\right)=\{\begin{array}{c} bp_{0}\left(x_{0}\right),X_{0}=\left\{x_{0}\right\}\\ 1-b,X_{0}=\emptyset \end{array}$$

where *b* ∈ [0,1] represents the probability of the target existing initially, which is named as initial existence probability.

The probability of detection is *P_D_* < 1. The sensor measurement model is:

(28) $$g\left(Z_{k}|X_{k}=\left\{x_{k}\right\}\right)=\{\begin{array}{c} P_{d}\xi \left(z_{k}|x_{k}\right),\;\;\;Z_{k}=\left\{z_{k}\right\}\\ 1-P_{d},\;\;Z_{k}=\emptyset \end{array}$$

(29) $$g\left(Z_{k}|X_{k}=\emptyset \right)=\{\begin{array}{c} 0,\;\;Z_{k}=\left\{z_{k}\right\}\\ 1,\;\;Z_{k}=\emptyset \end{array}$$

where *ξ*(**z***_k_*|**x***_k_*) is the measurement likelihood when the target is existing and detected, which can be calculated by (2). The measurement likelihood in (28) indicates there is some uncertainty in detection, and (29) means there is no false observation.

### 3.3. Random Finite Set Bound

In [20], the authors worked on the problem of calculating the PCRLBs for the cases of *P_D_* < 1 and *P_FA_* = 0. They proposed the possible time-sequence of observation-sets is given as follows:

(30) $$\Theta_{k,n}=\left\{Z_{1,n},Z_{2,n},\cdots ,Z_{k,n}\right\}$$

where *Z_k,n_* denotes the measurement is empty or not for sequence number *n*, at time-step *k*, and thus, *n* = 1,2,⋯,2*^k^*.

When *k* = 1:

(31) $$\Theta_{1,1}=\emptyset ,\Theta_{1,2}=\left\{z_{1}\right\};$$

When *k* ≥ 2, the Θ*_k_*_+1,_*_n_* is designed as follows:

(32) $$\begin{array}{l} \Theta_{k+1,n}\\ =\{\begin{array}{cc} \left\{\Theta_{k,n},\emptyset \right\}, & 1\leq n\leq 2^{k}\\ \left\{\Theta_{k,n-2^{k}},\left\{z_{k+1}\right\}\right\}, & 2^{k}+1\leq n\leq 2^{k+1} \end{array}\\ =\{\begin{array}{cc} \left\{\Theta_{k-1,n},\emptyset ,\emptyset \right\}, & 1\leq n\leq 2^{k-1}\\ \left\{\Theta_{k-1,n-2^{k-1}},\left\{z_{k}\right\},\emptyset \right\}, & 2^{k-1}+1\leq n\leq 2^{k}\\ \left\{\Theta_{k-1,n-2^{k}},\emptyset ,\left\{z_{k+1}\right\}\right\}, & 2^{k}+1\leq n\leq 2^{k}+2^{k-1}\\ \left\{\Theta_{k-1,n-2^{k}-2^{k-1}},\left\{z_{k}\right\},\left\{z_{k+1}\right\}\right\}, & 2^{k}+2^{k-1}+1\leq n\leq 2^{k+1} \end{array} \end{array}$$

For certain time-sequence of observation-sets Θ_*k*,*n*_, the error bound Σ_*k*,*n*_ is as follows:

(33) $$\Sigma_{k,n}=\int \cdots \int C_{k,n}p\left(X_{k},\Theta_{k,n}\right)\delta X_{k}\delta Z_{1,n}\cdots \delta Z_{k,n}\geq P_{k,n}=J_{k,n}^{-1}$$

where:

(34) $$C_{k,n}=e\left(X_{k},{\hat{X}}_{k}\left(\Theta_{k,n}\right)\right)e{\left(X_{k},{\hat{X}}_{k}\left(\Theta_{k,n}\right)\right)}^{T}$$

**P***_k,n_* is the bound and **J***_k,n_* is the FIM, for time-sequence of observation-sets Θ_*k*,*n*_.

Let us denote the error bound between two sets *X_k_* and the estimation *X̂_k_*(*Z*_1_ ⋯ *Z_k_*) is $\Sigma_{k}=\sum_{n=1}^{2^{k}}\Sigma_{k,n}$. Hence, from (33), as introduced in [20], the error limit calculated within the framework of the finite set statistics is denoted as **P***_k_*(*RFS*), and is given by:

(35) $$\Sigma_{k}\geq \sum_{n=1}^{2^{k}}P_{k,n}=P_{k}\left(\mathrm{RFS}\right)$$

As indicated in (35), in order to calculate the recursive form of **P***_k_*(*RFS*), we should obtain the recursive **P***_k,n_*. As the proof in [20], the performance bound could be represented as certain kind of multivariate functions of some time-based auxiliary elements as:

When time-step *k* ≥ 0, *n* = 1,⋯,2*^k^*^+ 1^, the bounds sequence **P***_k_*_+ 1,*n*_ obeys the recursion:

(36) $$P_{k+1,n}=\{\begin{array}{cc} {\tilde{P}}_{k+1,n}, & 1\leq n\leq 2^{k}\\ {\left[J_{k+1,n}\right]}^{-1}*\text{Pr}\left(\Theta_{k,n-2^{k}},Z_{k+1}\neq \emptyset \right), & 2^{k}+1\leq n\leq 2^{k+1} \end{array}$$

(37) $${\tilde{P}}_{k+1,n}=\{\begin{array}{ll} P_{k+1,n}^{*}, & \mathrm{trace}\left(P_{k+1,n}^{*}\right)\leq \mathrm{trace}\left(P_{k+1,n}^{**}\right)\\ P_{k+1,n}^{**}, & \mathrm{trace}\left(P_{k+1,n}^{*}\right)>\mathrm{trace}\left(P_{k+1,n}^{**}\right) \end{array}$$

where:

(38) $$P_{k+1,n}^{*}=e_{1}e_{1}^{T}\left(\text{Pr}\left(\Theta_{k,n},Z_{k+1}=\emptyset \right)-\rho_{k+1,n}\right)$$

(39) $$P_{k+1,n}^{**}=e_{0}e_{0}^{T}\rho_{k+1,n}+{\left[J_{k+1,n}\right]}^{-1}*\text{Pr}\left(\Theta_{k,n},Z_{k+1}=\emptyset \right)$$

(40) $$\rho_{k+1,n}=\int \ldots \int p\left(X_{k+1}=\emptyset ,\Theta_{k,n},Z_{k+1,n}=\emptyset \right)\delta Z_{1,n}\cdots \delta Z_{k,n}$$

As defined in (32), the measurement is empty *Z_k_*_+1_ = ∅ corresponds to the sequence number 1 ≤ *n* ≤ 2*^k^*, at time step *k* + 1. The reason of (37) is the estimator can have two possible assignments for the case where the measurement *Z_k_*_+1_ = ∅, which are:

(41) $${\hat{X}}_{k+1}\left(\Theta_{k,n},Z_{k+1,n}=\emptyset \right)={\tilde{x}}_{k+1}$$

and:

(42) $${\hat{X}}_{k+1}\left(\Theta_{k,n},Z_{k+1,n}=\emptyset \right)=\emptyset$$

where estimation **x̃***_k_*_+1_ is the prediction, whose bound is calculated by (14), when *Z_k_*_+1,_*_n_* = ∅. Therefore, the lower bound on the estimate error will be the minimum of the bounds on the two estimations.

Recursive relationships of all above auxiliary elements are derived rigorously in [20]. First, according (14) and (32), when time-step *k* ≥ 0, *n* = 2*^k^*^+ 1^, the FIM **J***_k_*_+ 1,*n*_ obey the recursion:

(43) $$\begin{array}{l} J_{k+1,n}=\\ \{\begin{array}{c} Q_{k}^{-1}-Q_{k}^{-1}E\left\{F_{k}\right\}{\left[J_{k}+E\left\{F_{k}^{T}Q_{k}^{-1}F_{k}\right\}\right]}^{-1}E\left\{F_{k}\right\}Q_{k}^{-1},\\ 1\leq n\leq 2^{k}\\ Q_{k}^{-1}-Q_{k}^{-1}E\left\{F_{k}\right\}\left[J_{k}+E{\left\{F_{k}^{T}Q_{k}^{-1}F_{k}\right\}}^{-1}\right]E\left\{F_{k}\right\}Q_{k}^{-1}+E\left\{H_{k+1}^{T}R_{k+1}^{-1}H_{k+1}\right\},\\ 2^{k}+1\leq n\leq 2^{k+1} \end{array} \end{array}$$

Second, when *k* = 0:

(44) $$\text{Pr}\left(\Theta_{0,1},Z_{1}=\emptyset \right)=\text{Pr}\left(\Theta_{1,1}\right)=1-bP_{D}$$

(45) $$\text{Pr}\left(\Theta_{0,1},Z\neq \emptyset \right)=\text{Pr}\left(\Theta_{1,2}\right)=bP_{D}$$

(46) $$\rho_{1,1}=1-b$$

When *k* ≥ 1, from (36), the problem of recursion of **P***_k,n_* reduces to the recursion of *p_k_*_+ 1_*_,n_* and Pr(Θ*_k_*_+1,_*_n_*). When the time step *k* ≥ 1, *n* = 1,⋯,2*^k^*^+ 1^:

(47) $$\text{Pr}\left(\Theta_{k+1,n}\right)=\{\begin{array}{cc} \text{Pr}\left(\Theta_{k,n}\right)p\left(Z_{k+1,n}=\emptyset |\Theta_{k,n}\right), & 1\leq n\leq 2^{k-1}\\ \text{Pr}\left(\Theta_{k,n}\right)\left(1-rP_{D}\right), & 2^{k-1}+1\leq n\leq 2^{k}\\ \text{Pr}\left(\Theta_{k,n-2^{k}}\right)\left[1-p\left(Z_{k+1,n}=\emptyset |\Theta_{k,n-2^{k}}\right)\right], & 2^{k}+1\leq n\leq 2^{k}+2^{k-1}\\ \text{Pr}\left(\Theta_{k,n-2^{k}}\right)rP_{D}, & 2^{k}+2^{k-1}+1\leq n\leq 2^{k+1} \end{array}$$

When *k* ≥ 1:

(48) $$\rho_{k+1,n}=\text{Pr}\left(\Theta_{k,n}\right)\int \ldots \int \frac{p\left(Z_{k+1,n}=\emptyset |\Theta_{k,n}\right)-\left(1-P_{D}\right)}{P_{D}}\delta Z_{1,n}\cdots \delta Z_{k,n},1\leq n\leq 2^{k}$$

Finally, from (47) and (48), the key of the recursion of *p_k_*_+ 1,*n*_ and Pr(Θ_*k*+1,*n*_) is the recursion of *p*(*Z*_*k*+1,*n*_ = ∅|Θ_*k*,*n*_), 1 ≤ *n* ≤ 2*^k^*.

When *k* ≥ 1:

(49) $$\begin{array}{l} p\left(Z_{k+1,n}=\emptyset |\Theta_{k,n}\right)\\ =\{\begin{array}{cc} 1-rP_{D}+\left(2r-1\right)\frac{p\left(Z_{k,n}=\emptyset |\Theta_{k-1,n}\right)-\left(1-P_{D}\right)}{p\left(Z_{k,n}=\emptyset |\Theta_{k-1,n}\right)}, & 1\leq n\leq 2^{k-1}\\ 1-rP_{D}, & 2^{k-1}+1\leq n\leq 2^{k} \end{array}\\ =\Gamma \left(p\left(Z_{k,n}=\emptyset |\Theta_{k-1,n}\right)\right) \end{array}$$

Then the recursive form of bound recursions is turned up, and we obtain the recursive **P***_k,n_*.

## 4. Comparison of Three Bounds

This section presents a comparison of the RFS bound, the IRF PCRLB and the ENUM PCRLB, as introduced in the previous sections. This problem is discussed in two cases. Section 4.1 introduces the first case, where the target exists from the beginning to the end. Section 4.2 is devoted to the other case, where the new targets might appear and existing targets could disappear. In Section 4.1, we firstly deduce a comparable form of the RFS bound. Then, this form of bound is applied to compare with ENUM PCRLB in Section 4.1.2, while it is also used to compare with IRF PCRLB in Section 4.1.3. In Section 4.2, these three bounds are compared directly both quantitatively and qualitatively.

### 4.1. Case I: Target Exists form the Beginning to the End

#### 4.1.1. Comparable form of Random Set Estimation Bound

All PCRLBs are based on the assumption that the target always exists. In order to compare the bound in [20] to PCRLB, we firstly introduce the relationship between the bound and the probability of the state set is not empty:

*Proposition I*: if both the

Condition1:

(50) $$e_{1}=e_{0}={\left[\begin{array}{cccc} e_{1,1} & e_{2,1} & \ldots & e_{m,1} \end{array}\right]}^{T}$$

and

Condition2:

(51) $$\sum_{i=1}^{m}e_{i,1}^{2}>>\mathrm{trace}\left(J_{k+1,n}^{-1}\right)>0$$

are satisfied, the bound calculated in the framework of random finite set is as follows:

(52) $$\begin{array}{l} {\tilde{P}}_{k+1,n}=\\ \{\begin{array}{ll} P_{k+1,n}^{*}, & \int \cdots \int p\left(X_{k+1}\neq \emptyset ,\Theta_{k,n},Z_{k+1,n}=\emptyset \right)\delta Z_{1,n}\cdots \delta Z_{k,n}\leq \int \cdots \int p\left(X_{k+1}=\emptyset ,\Theta_{k,n},Z_{k+1,n}=\emptyset \right)\delta Z_{1,n}\cdots \delta Z_{k,n}\\ P_{k+1,n}^{**}, & \int \cdots \int p\left(X_{k+1}\neq \emptyset ,\Theta_{k,n},Z_{k+1,n}=\emptyset \right)\delta Z_{1,n}\cdots \delta Z_{k,n}>\int \cdots \int p\left(X_{k+1}=\emptyset ,\Theta_{k,n},Z_{k+1,n}=\emptyset \right)\delta Z_{1,n}\cdots \delta Z_{k,n} \end{array} \end{array}$$

where:

Proof:

Assume that:

(53) $$\int \cdots \int p\left(X_{k+1}\neq \emptyset ,\Theta_{k,n},Z_{k+1,n}=\emptyset \right)\delta Z_{1,n}\cdots \delta Z_{k,n}>\int \cdots \int p\left(X_{k+1}=\emptyset ,\Theta_{k,n},Z_{k+1,n}=\emptyset \right)\delta Z_{1,n}\cdots \delta Z_{k,n}$$

As the number of the scans of measurements increases, Condition 2 should be met easily. Then from (53), with the Condition 2, we can deduce that:

(54) $$\begin{array}{l} \left(\sum_{1}^{m}e_{i,1}^{2}-\mathrm{trace}\left(J_{k+1,n}^{-1}\right)\right)\int \cdots \int p\left(X_{k+1}\neq \emptyset ,\Theta_{k,n},Z_{k+1,n}=\emptyset \right)\delta Z_{1,n}\cdots \delta Z_{k,n}\\ >\left(\sum_{1}^{m}e_{i,1}^{2}+\mathrm{trace}\left(J_{k+1,n}^{-1}\right)\right)\int \cdots \int p\left(X_{k+1}=\emptyset ,\Theta_{k,n},Z_{k+1,n}=\emptyset \right)\delta Z_{1,n}\cdots \delta Z_{k,n} \end{array}$$

then:

(55) $$\begin{array}{l} \sum_{1}^{m}e_{i,1}^{2}*\int \cdots \int p\left(X_{k+1}\neq \emptyset ,\Theta_{k,n},Z_{k+1,n}=\emptyset \right)\delta Z_{1,n}\cdots \delta Z_{k,n}>\sum_{1}^{m}e_{i,1}^{2}*\int \cdots \int p\left(X_{k+1}=\emptyset ,\Theta_{k,n},Z_{k+1,n}=\emptyset \right)\delta Z_{1,n}\cdots \delta Z_{k,n}\\ +\mathrm{trace}\left(J_{k+1,n}^{-1}\right)\left(\begin{array}{l} \int \cdots \int p\left(X_{k+1}=\emptyset ,\Theta_{k,n},Z_{k+1,n}=\emptyset \right)\delta Z_{1,n}\cdots \delta Z_{k,n}+\\ \int \cdots \int p\left(X_{k+1}\neq \emptyset ,\Theta_{k,n},Z_{k+1,n}=\emptyset \right)\delta Z_{1,n}\cdots \delta Z_{k,n} \end{array}\right) \end{array}$$

Combining the definition of *p_k_*_+ 1_*_,n_* in (40), it is easy to derive that:

(56) $$\left(\text{Pr}\left(\Theta_{k,n},Z_{k+1}=\emptyset \right)-\rho_{k+1,n}\right)*\sum_{1}^{m}e_{i,1}^{2}>\rho_{k+1,n}*\sum_{1}^{m}e_{i,1}^{2}+\mathrm{trace}\left(J_{k+1,n}^{-1}\right)*\text{Pr}\left(\Theta_{k,n},Z_{k+1}=\emptyset \right)$$

Because of Condition 1 that **e**_1_ = **e**_0_ = [*e*_1,1_  *e*_2,1_   ⋯   *e_m_*_,1_]*^T^*, it is clear that:

(57) $$\mathrm{trace}\left(e_{1}e_{1}^{T}\left(\text{Pr}\left(\Theta_{k,n},Z_{k+1}=\emptyset \right)-\rho_{k+1,n}\right)\right)>\mathrm{trace}\left(e_{0}e_{0}^{T}\rho_{k+1,n}+{\left[J_{k+1,n}\right]}^{-1}*\text{Pr}\left(\Theta_{k,n},Z_{k+1}=\emptyset \right)\right)$$

As defined in (38) and (39), the inequality (57) means that:

(58) $$\mathrm{trace}\left(P_{k+1,n}^{*}\right)>\mathrm{trace}\left(P_{k+1,n}^{**}\right)$$

Then, the bound for a time-sequence of observation-sets Θ*_k,n_* is:

(59) $${\tilde{P}}_{k+1,n}=P_{k+1,n}^{**}$$

Proposition I denotes that, when the probability of not empty state set is more than which of empty, the bound is in the form that ${\tilde{P}}_{k+1,n}=e_{0}e_{0}^{T}\rho_{k+1,n}+{\left[J_{k+1,n}\right]}^{-1}*\text{Pr}\left(\Theta_{k,n},Z_{k+1}=\emptyset \right),1\leq n\leq 2^{k}$.

#### 4.1.2. Comparison of Enumeration PCRLB and Random Finite Set Bound

The calculation of PCRLB is based on the assumption that the target exists from the beginning to the end. Moreover, there is no false alarm. For the RFS bound, as defined in (40), this assumption means that the state set is impossible to be empty:

(60) $$\begin{array}{l} \rho_{k+1,n}=\int \cdots \int p\left(X_{k+1}=\emptyset ,\Theta_{k,n},Z_{k+1,n}=\emptyset \right)\delta Z_{1,n}\cdots \delta Z_{k,n}\\ =\int \cdots \int p\left(X_{k+1}=\emptyset ,\Theta_{k,n}\right)\delta Z_{1,n}\cdots \delta Z_{k,n}\\ =0 \end{array}$$

Therefore, the bound calculated by (37) is simplified as:

(61) $${\tilde{P}}_{k+1,n}={\left[J_{k+1,n}\right]}^{-1}*\text{Pr}\left(\Theta_{k,n},Z_{k+1}=\emptyset \right),1\leq n\leq 2^{k}$$

Then, the calculation of **P***_k_*_+ 1_(RFS) in (35):

(62) $$\begin{array}{l} P_{k+1}\left(\text{RFS}\right)\\ =\sum_{n=1}^{2^{k}}P_{k,n}\\ =\sum_{n=1}^{2^{k}}\left\{{\left[Q_{k}^{-1}-Q_{k}^{-1}E\left\{F_{k}\right\}{\left[J_{k}+E\left\{F_{k}^{T}Q_{k}^{-1}F_{k}\right\}\right]}^{-1}E\left\{F_{k}\right\}Q_{k}^{-1}\right]}^{-1}\text{Pr}\left(\Theta_{k,n},Z_{k+1}=\emptyset \right)\right\}\\ +\sum_{n=2^{k}+1}^{2^{k+1}}\left\{{\left[Q_{k}^{-1}+E\left\{H_{k+1}^{T}R_{k+1}^{-1}H_{k+1}\right\}-Q_{k}^{-1}E\left\{F_{k}\right\}{\left[J_{k}+E\left\{F_{k}^{T}Q_{k}^{-1}F_{k}\right\}\right]}^{-1}E\left\{F_{k}\right\}Q_{k}^{-1}\right]}^{-1}\text{Pr}\left(\Theta_{k,n-2^{k}},Z_{k+1}\neq \emptyset \right)\right\} \end{array}$$

As indicated in (47), the probability Pr(Θ*_k_*_+1,_*_n_*) can be divided into four parts, and thus the bound can be written as a sum of four parts:

(63) $$\begin{array}{l} P_{k+1}\left(\text{RFS}\right)\\ =\sum_{n=1}^{2^{k-1}}\left\{{\left[Q_{k}^{-1}-Q_{k}^{-1}E\left\{F_{k}\right\}{\left[J_{k}+E\left\{F_{k}^{T}Q_{k}^{-1}F_{k}\right\}\right]}^{-1}E\left\{F_{k}\right\}Q_{k}^{-1}\right]}^{-1}\text{Pr}\left(\Theta_{k,n}\right)p\left(Z_{k+1,n}=\emptyset |\Theta_{k,n}\right)\right\}\\ +\sum_{n=2^{k-1}+1}^{2^{k}}\left\{{\left[Q_{k}^{-1}-Q_{k}^{-1}E\left\{F_{k}\right\}{\left[J_{k}+E\left\{F_{k}^{T}Q_{k}^{-1}F_{k}\right\}\right]}^{-1}E\left\{F_{k}\right\}Q_{k}^{-1}\right]}^{-1}\text{Pr}\left(\Theta_{k,n}\right)\left(1-rP_{D}\right)\right\}\\ +\sum_{n=2^{k}+1}^{2^{k}+2^{k-1}}\left\{\begin{array}{l} {\left[Q_{k}^{-1}-Q_{k}^{-1}E\left\{F_{k}\right\}{\left[J_{k}+E\left\{F_{k}^{T}Q_{k}^{-1}F_{k}\right\}\right]}^{-1}E\left\{F_{k}\right\}Q_{k}^{-1}+E\left\{H_{k+1}^{T}R_{k+1}^{-1}H_{k+1}\right\}\right]}^{-1}\\ \text{Pr}\left(\Theta_{k,n-2^{k}}\right)\left(1-p\left(Z_{k+1,n}=\emptyset |\Theta_{k-2^{k}}\right)\right) \end{array}\right\}\\ +\sum_{n=2^{k}+2^{k-1}+1}^{2^{k+1}}\left\{\begin{array}{l} {\left[Q_{k}^{-1}-Q_{k}^{-1}E\left\{F_{k}\right\}{\left[J_{k}+E\left\{F_{k}^{T}Q_{k}^{-1}F_{k}\right\}\right]}^{-1}E\left\{F_{k}\right\}Q_{k}^{-1}+E\left\{H_{k+1}^{T}R_{k+1}^{-1}H_{k+1}\right\}\right]}^{-1}\\ \text{Pr}\left(\Theta_{k,n-2^{k}}\right)rP_{D} \end{array}\right\} \end{array}$$

Although we have the recursion of the conditional probability *p*(*Z_k_*_+1,_*_n_* = ∅|Θ*_k,n_*) as (49), the relationship between this conditional probability and the model parameters should be more clear, in order to compare this bound to the PCRLB. Such relationship is denoted as follows:

At time step *k* + 1, for the sequence number 1 ≤ *n* ≤ 2*^k^*^− 1^, if it is satisfied that:

Condition 3: the maintenance probability is the unity as:

(64) r=1

Condition 4: the initial probability is the unity as:

(65) b=1

Then the conditional probability *p*(*Z_k_*_+1,_*_n_* = ∅|Θ*_k,n_*) is as follows:

(66) $$p\left(Z_{k+1,n}=\emptyset |\Theta_{k,n}\right)=1-P_{D}$$

Proof:

For the case where the false alarm probability *P_FA_* = 0, under the assumption that there is always one target, the probability of no measurement is equal to the missed—detection probability, *i.e*., 1 − *P_D_*.

Additionally, when *p*(*Z_k_*_+1,*n*_ = ∅|Θ*_k,n_*) = 1 − *P_D_*, using (48), it is clear that *p_k_*_+ 1,*n*_ = 0,1 ≤ *n* ≤ 2*^k^*.

From (63) and (66), we obtain that:

(67) $$\begin{array}{l} P_{k+1}\left(\text{RFS}\right)\\ =\sum_{n=1}^{2^{k}}\left\{{\left[Q_{k}^{-1}-Q_{k}^{-1}E\left\{F_{k}\right\}{\left[J_{k}+E\left\{F_{k}^{T}Q_{k}^{-1}F_{k}\right\}\right]}^{-1}E\left\{F_{k}\right\}Q_{k}^{-1}\right]}^{-1}\text{Pr}\left(\Theta_{k,n}\right)\left(1-P_{D}\right)\right\}\\ +\sum_{n=2^{k}+1}^{2^{k+1}}\left\{{\left[Q_{k}^{-1}+E\left\{H_{k+1}^{T}R_{k+1}^{-1}H_{k+1}\right\}-Q_{k}^{-1}E\left\{F_{k}\right\}{\left[J_{k}+E\left\{F_{k}^{T}Q_{k}^{-1}F_{k}\right\}\right]}^{-1}E\left\{F_{k}\right\}Q_{k}^{-1}\right]}^{-1}\text{Pr}\left(\Theta_{k,n-2^{k}}\right)P_{D}\right\} \end{array}$$

As in [11], the probability of occurrence of a particular detection/miss sequence Pr(Θ*_k,n_*) is given by:

(68) $$\text{Pr}\left(\Theta_{k,n}\right)=P_{D}^{\Delta_{k,n}}*{\left(1-P_{D}\right)}^{k-\Delta_{k,n}}$$

where the number of detections in a particular sequence Θ*_k,n_* is Δ*_k,n_*.

Rewrite the EUNM PCRLB in (14) and (16) as follows:

(69) $$\begin{array}{l} P_{k+1}\left(ENUM\right)=\\ \sum_{n=1}^{2^{k}}\left\{{\left[Q_{k}^{-1}-Q_{k}^{-1}E\left\{F_{k}\right\}{\left[J_{k}+E\left\{F_{k}^{T}Q_{k}^{-1}F_{k}\right\}\right]}^{-1}E\left\{F_{k}\right\}Q_{k}^{-1}\right]}^{-1}*\text{Pr}\left(\Theta_{k,n},Z_{k+1}=\emptyset \right)\right\}\\ +\sum_{n=2^{k}+1}^{2^{k+1}}\left\{{\left[Q_{k}^{-1}-Q_{k}^{-1}E\left\{F_{k}\right\}{\left[J_{k}+E\left\{F_{k}^{T}Q_{k}^{-1}F_{k}\right\}\right]}^{-1}E\left\{F_{k}\right\}Q_{k}^{-1}+E\left\{H_{k+1}^{T}R_{k+1}^{-1}H_{k+1}\right\}\right]}^{-1}*\text{Pr}\left(\Theta_{k,n-2^{k}},Z_{k+1}\neq \emptyset \right)\right\}\\ =\sum_{n=1}^{2^{k}}\left\{{\left[Q_{k}^{-1}-Q_{k}^{-1}E\left\{F_{k}\right\}{\left[J_{k}+E\left\{F_{k}^{T}Q_{k}^{-1}F_{k}\right\}\right]}^{-1}E\left\{F_{k}\right\}Q_{k}^{-1}\right]}^{-1}\text{Pr}\left(\Theta_{k,n}\right)\left(1-P_{D}\right)\right\}\\ +\sum_{n=2^{k}+1}^{2^{k+1}}\left\{{\left[Q_{k}^{-1}-Q_{k}^{-1}E\left\{F_{k}\right\}{\left[J_{k}+E\left\{F_{k}^{T}Q_{k}^{-1}F_{k}\right\}\right]}^{-1}E\left\{F_{k}\right\}Q_{k}^{-1}+E\left\{H_{k+1}^{T}R_{k+1}^{-1}H_{k+1}\right\}\right]}^{-1}\text{Pr}\left(\Theta_{k,n-2^{k}}\right)P_{D}\right\} \end{array}$$

Therefore, we can now show the relationship between *P_k_*_+ 1_(*RFS*) and *P_k_*_+ 1_(*EUNM*) as follows:

*Proposition II*: For the case of *P_D_* < 1 and *P_FA_* = 0, relying on Condtion 1, 2, 3 and 4, the following result is true:

(70) $$P_{k}\left(RFS\right)=P_{k}\left(ENUM\right)$$

*Proof*: By Proposition I and *p*(*Z_k_*_+1,_*_n_* = ∅|Θ*_k,n_*) = 1 − *P_D_*, we obtain the form of RFS bound as (67). (70) is obvious from (67) and (69). This means that **P***_k_*(*RFS*) reduces to **P***_k_*(*ENUM*) with time, when the target exists from the beginning to the end.

#### 4.1.3. Comparison of Information Reduction Factor PCRLB and Random Finite Set Bound

In [12], for the case where *P_D_* < 1 and *P_FA_* = 0, the authors indicated the relationship between the ENUM PCRLB and IRF PCRLB as:

(71) $$P_{k}\left(ENUM\right)>P_{k}\left(IRF\right)$$

when the following conditions are met:

Condition 5:

(72) $$J_{k}\;\text{is positive definite}$$

Condition 6:

(73) $$J_{k}\left(S_{i}\right)\;\text{is}\;\text{positive}\;\text{definite}\;\text{for}\;\text{all}\;S_{i}$$

It is obvious that Condition 5 and Condition 6 are satisfied when we get the recursive bound for random finite set in [20].

Therefore, for the case where *P_D_* < 1 and *P_FA_* = 0, relying on Condtion 1, 2, 3, 4, 5 and 6, the following result is true:

(74) $$P_{k}\left(RFS\right)>P_{k}\left(IRF\right)$$

All above proofs are in the case of *P_D_* < 1 and *P_FA_* = 0.

When the probability of detection is zero or unity, such as *P_D_* = 1 or *P_D_* = 0, as indicated in [12], all the three bounds are identical:

(75) $$P_{k}\left(RFS\right)=P_{k}\left(ENUM\right)=P_{k}\left(IRF\right)$$

where the Conditions 1, 2, 3 and 4 should be satisfied.

### 4.2. Case II: Target Appears or Disappears with Probability

All PCRLBs are based on the assumption that the target exists from the beginning to the end. However, the existence of the target is difficult to be deterministic, with phenomena such as target spawning, new-born targets or disappearing ones. This problem finds lots of applications in defense and surveillance [13,14], where it is unknown whether the target exists or not and the aim is to determine the existence of the target and its state from the sensor measurement [18]. In the traditional framework, the problem is solved by data association, but data association is complicated and is still an open question for some applications. For example, when a target has disappeared but there is a false detection, we might mistakenly use the PCRLB to limit its error.

In fact, there are many tracking algorithms, such as [15], [16] and [17], based on RFS, and they have generally ignored the issue of data association. For these algorithms, the state of targets and the sensor measurements should be modeled as RFS. Moreover, the target uncertainties, such as spawning, new-born and disappearing targets, are all described by different RFSs. At the same time, the uncertainties of observation, such as *P_D_* < 1 and *P_FA_* > 0, are also modeled by RFSs. Therefore, the bound deduced in the framework of the finite set statistics is more suitable than PCRLB, when there are abundant uncertainties of target dynamics and sensors detection.

Although the RFS bound **P***_k_*(*RFS*) is different with ENUM PCRLB **P***_k_*(*ENUM*), the comparison between them is difficult. On one hand, as discussed in Section 4.2, if the target always exists, RFS bound **P***_k_*(*RFS*) is similar with the **P***_k_*(*ENUM*), when four conditions are met, but in this case, the meaning of RFS bound **P***_k_*(*RFS*) is reduced. On the other hand, when targets might appear or disappear, RFS bound **P***_k_*(*RFS*) is different with PCRLB, and thus the meaning of **P***_k_*(*RFS*) is significant. Unfortunately, when targets appear or disappear with certain probability, **P***_k_*(*RFS*) and **P***_k_*(*ENUM*) cannot be compared directly by quantitatively.

The quantitative comparison is difficult. The reason is that the RFS bound and ENUM PCRLB are calculated by different models. The model for calculating **P***_k_*(*ENUM*) is given in Section 2.1, while the model for **P***_k_*(*RFS*) is shown in Section 3.2. For the models in Sections 2.1 and 3.2, though the state dynamics are the same and the measurement likelihood functions are also similar, when the target exists and it is detected, these two models are still different. The reason is that the model of **P***_k_*(*RFS*) can take the target appearance or disappearance into account, but the model of **P***_k_*(*ENUM*) cannot. Such a difference leads to that the recursions of Pr(Θ*_k,n_*) are different. For example, to obtain **P***_k_*(*RFS*), the probability of certain time-sequence of observation-sets Pr(Θ*_k,n_*) is calculated by (47) and (49), which is a function of the maintenance probability *r*, the detection probability *P_D_* and the conditional probability at *k* − 1 step *p*(*Z_k_*_−1,_*_n_* = ∅|Θ_*k*−2,*n*_). But, when **P***_k_*(*ENUM*) is calculated, the probability Pr(Θ*_k,n_*) is only decided by the detection probability *P_D_* as in (68), and moreover, it is not recursive.

The qualitative comparison analysis is also difficult. If we only considered the uncertainty brought by new-born and disappearing targets, the RFS bound **P***_k_*(*RFS*) should be bigger than PCRLB. However, when sensor measurement is empty, the estimation can possibly be empty. In this case, if the target state is empty, the error is zero, as defined in (24). Therefore, the uncertainty brought by measurement is reduced, and thus the **P***_k_*(*RFS*) might be smaller than PCRLB. In conclusion, the uncertainty of target existence and the method of calculating estimation error have the opposite effect on the RFS bound **P***_k_*(*RFS*).

Although it is hard to determine that the bound **P***_k_*(*RFS*) is more than PCRLB or not, for targets appearing or disappearing, there are abundant application cases, which could show the relationship between the bound **P***_k_*(*RFS*) and PCRLB.

## 5. Application Examples

In this section, two examples are used to illustrate previous results. In the first case, we give an example to show how the four conditions in Section 4 influence the relationship between the RFS bound **P***_k_*(*RFS*) and PCRLB, when the target exists from the beginning to the end. Then, by both the two cases, we discuss the relationship between **P***_k_*(*RFS*) and PCRLB with varying the parameters of RFS model, when targets might appear or disappear.

### 5.1. Ballistic Object Tracking on Re-Entry

Online estimation of the kinematic state of a ballistic object re-entering the atmosphere is an important problem. This section used a simple motion model as in [12]. In order to simplify the model, we use Euler approximation with a very small integration step *τ*<<*T* to get the dynamic equation:

(76) $$x_{l+1}=\Phi x_{l}-G\left[\delta \left(x_{l}\right)-g\right]$$

where *T* is the sampling scan and *T*/*τ* = *L* is an integer, so the relationship between discrete-time indices *k* and *l* is *k* = ⌊*l/L*⌋. The state vector is **x***_l_* = [*h_l_v_l_β_l_*], where *h_l_* is the object height, *v_l_* is the velocity and *β_l_* is the ballistic coefficient. Other parameters in the state equation are as follows:

(77) $$\Phi =\left[\begin{array}{ccc} 1 & -\tau & 0\\ 0 & 1 & 0\\ 0 & 0 & 1 \end{array}\right]$$

(78) $$G=\left[\begin{array}{ccc} 0 & \tau & 0 \end{array}\right]$$

(79) $$\delta \left(x_{l}\right)=\frac{g\rho_{l}v_{l}^{2}}{2\beta_{l}}$$

The exponentially decaying model of air density is adopted as *ρ_l_* = *γ*exp{−*ηh_l_*}, where *γ* = 1.745 and *η* = 1.49*10^−4^. The covariance matrix **Q***_l_* of process noise is given by:

(80) $$Q_{l}=\left[\begin{array}{ccc} \tau^{3}/3 & \tau^{2}/2 & 0\\ \tau^{2}/2 & \tau & 0\\ 0 & 0 & \tau \end{array}\right]$$

The radar measures the height of target with *z_k_* and at regular intervals of *T*s. The measurement equation is:

(81) $$z_{k}=Hx_{k}+v_{k}$$

Where *H* = [1 0 0], and the variance of measurement noise is given by $R=\sigma_{r}^{2}$.

The prior distribution is assumed Gaussian with covariance, and the initial FIM is calculated as in (12):

(82) $$P_{0}=\left[\begin{array}{ccc} \sigma_{r}^{2} & \sigma_{r}^{2}/T & 0\\ \sigma_{r}^{2}/T & 2\sigma_{r}^{2}/T & 0\\ 0 & 0 & \sigma_{\beta }^{2} \end{array}\right]$$

where $\sigma_{\beta }^{2}$ is selected to cover all possible values of the ballistic coefficient.

The Jacobian defined by (9) is given by:

(83) $$F_{l}=\left[\begin{array}{ccc} 1 & -\tau & 0\\ f_{21}\tau & 1-f_{22}\tau & f_{23}\tau \\ 0 & 0 & 1 \end{array}\right]$$

where:

(84) $$f_{21}=\frac{\eta g\rho_{l}v_{l}^{2}}{2\beta_{l}}$$

(85) $$f_{22}=\frac{g\rho_{l}v_{l}}{\beta_{l}}$$

(86) $$f_{23}=\frac{g\rho_{l}v_{l}^{2}}{2\beta_{l}^{2}}$$

Following parameters are applied in this example. The initial target state vector **x**_0_ = [55,000 m 300 m/s, 22,500 kg/ms^2^]. The integration time *τ* = 0.1*s* and the sampling interval *T* = 1 s. The variances *σ_r_* = 300 m and *σ_β_* = 10,000 kg/ms^2^.

Here we set **e**_1_ = **e**_0_, and thus the Condition 1 is met. In this example, we always set *b* = 1 in (27). In other words, the Condition 4 is met. Condition 5 and 6 are also satisfied in this case.

**Figure 1.**
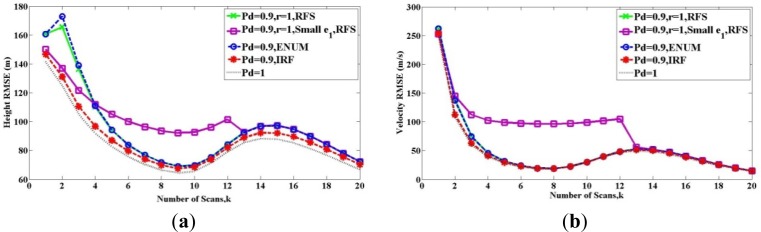
Comparisons of bounds when the target exists from the beginning to the end with different setting cardinality mismatches: (**a**) Height (**b**) Velocity.

**Figure 2.**
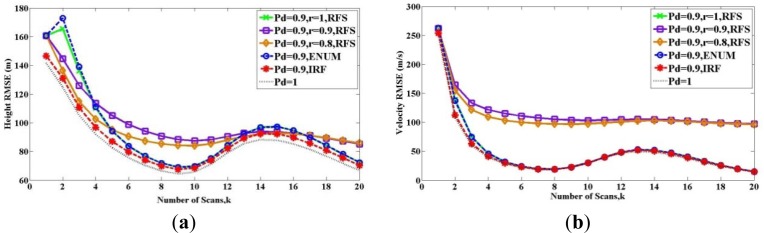
Comparisons of bounds when the target disappears with different maintenance probability r: (**a**) Height (**b**) Velocity.

Figures 1 and 2 show the root-mean-square error (RMSE) bound between two sets *X_k_* and *X̂_k_*(*Z*_1_ ⋯ *Z_k_*) in (a) height and (b) velocity for twenty scans, when the target exists from the beginning to the end. The solid lines show the RFS bound **P***_k_*(*RFS*). The dashed lines represent the PCRLBs, where **P***_k_*(*ENUM*) is shown by the blue one and **P***_k_*(*IRF*) is shown by the red one. All above bounds are calculated at *P_D_* = 0.9. The black dashed line is the bound for the detection probability *P_D_* = 1.

In Figure 1, the two solid lines show the bound based on RFS model **P***_k_*(*RFS*), and the maintenance probability maintains *r* = 1. This means the target exists from the first scan to the last one, and thus it meets the Condition 3.

When we compare the two solid lines, Figure 1 shows the influence of Condition 2. The green solid line is for the case where the Condition 2 is satisfied except in the initial steps, because we set **e**_1_**e**_1_*^T^* = **e**_0_**e**_0_*^T^* = *P*_0_. Therefore, as the scan number increases and measurements become more, the Condition 2 is satisfied. In this condition, we can see that **P***_k_*(*RFS*) shown by the green line is equal to **P***_k_*(*ENUM*), as discussed in Section 4.1. If there is **e**_1_**e**_1_*^T^* = **e**_0_**e**_0_*^T^* = 0.5*P*_0_, the Condition 2 is not met, as shown by the purple solid line. At the beginning, the purple line is below the green one, because its cardinality mismatches **e**_0_ and **e**_1_ are smaller. But after these scans, the purple line is more the green one. In conclusion, by the influence of wrong settings **e**_0_ and **e**_1_, **P***_k_*(*RFS*) shown by the purple line is not equal to **P***_k_*(*ENUM*), since the selection of **P̃***_k_*_+1,_*_n_* in (37) is mistaken. After 13 scans, they are equal. Although we were unable to prove the convergence of the two bounds, there is an intuitive explanation. Because the target exists from the beginning to the end, and the detection probability is high (*P_D_* = 0.9), with the scans increasing, the probability Pr(Θ_*k,n*−2^*k*^_, *Z_k_*_+1_ ≠ ∅) is much more bigger than the probability Pr(Θ_*k,n*−2^*k*^_, *Z_k_*_+1_ = ∅) in (36). It means the FIM [**J***_k_*_+1,_*_n_*]^−1^*Pr(Θ_*k,n*−2^*k*^_, *Z_k_*_+1_ ≠ ∅) provides more contribution than **P̃***_k_*_+1,_*_n_* to the calculation of RFS bound **P***_k_*(*RFS*). The wrongly setting **e**_0_ and **e**_1_ becomes less important with time, and eventually, both **P***_k_*(*RFS*)s shown by the green and purple lines become similar.

In Figure 2, the three solid lines show **P***_k_*(*RFS*), with setting **e**_1_**e**_1_*^T^* = **e**_0_**e**_0_*^T^* = *P*_0_. Thus the Condition 2 is satisfied as the scan number increases. When we compare the three solid lines, Figure 2 shows the influence of Condition 3. The green solid line is for the case where the existence of the target is deterministic (*r* = 1), we can see that **P***_k_*(*RFS*) shown by the green line is equal to **P***_k_*(*ENUM*), as discussed in Section 4.1. When *r* < 1 and *b* = 1, it means the target disappear with the probability 1 − *r*, the purple line and the yellow line are unequal to the green line or PCRLB **P***_k_*(*ENUM*). As discussed in Section 4.2, when the target might disappear, **P***_k_*(*RFS*) and **P***_k_*(*ENUM*) cannot be compared directly by quantitatively. The reason why RFS bounds and PCRLBs intersect with each other will be further discussed in the next example.

### 5.2. Bearings-Only Tracking

This example is similar to the bearings-only tracking case in [12]. This system can be applied in various sensors, such as electro-magnetic (EM) equipment, electronic warfare devices (ESM) and passive sonar [12].

The observer, named ownship, is a moving platform carrying sensor. Its state vector is denoted as $x_{k}^{o}={\left[\begin{array}{cccc} \chi_{k}^{o} & {\dot{\chi }}_{k}^{o} & \gamma_{k}^{o} & {\dot{\gamma }}_{k}^{o} \end{array}\right]}^{T}$ and assumed known. The target vector is denoted as $x_{k}^{t}={\left[\begin{array}{cccc} \chi_{k}^{t} & {\dot{\chi }}_{k}^{t} & \gamma_{k}^{t} & {\dot{\gamma }}_{k}^{t} \end{array}\right]}^{T}$. The relative state vector is defined as:

(87) $$x_{k}=x_{k}^{t}-x_{k}^{o}={\left[\begin{array}{cccc} \chi_{k} & {\dot{\chi }}_{k} & \gamma_{k} & {\dot{\gamma }}_{k} \end{array}\right]}^{T}$$

where (*χ_k_*, *γ_k_*) is the relative target position and (*χ̇_k_*, *γ̇_k_*) is its velocity. The dynamic equation is as follows:

(88) $$x_{k+1}=F_{k}x_{k}-U_{k,k+1}$$

where *F_k_* is denoted in:

(89) $$F_{k}=\left[\begin{array}{cccc} 1 & T & 0 & 0\\ 0 & 1 & 0 & 0\\ 0 & 0 & 1 & T\\ 0 & 0 & 0 & 1 \end{array}\right]$$

and the effect of a mismatch between the observer and the target motion model is accounted by :

(90) $$U_{k,k+1}=\left[\begin{array}{c} \chi_{k+1}^{o}-\chi_{k}^{o}-T{\dot{\chi }}_{k}^{o}\\ {\dot{\chi }}_{k+1}^{o}-{\dot{\chi }}_{k}^{o}\\ \gamma_{k+1}^{o}-\gamma_{k}^{o}-T{\dot{\gamma }}_{k}^{o}\\ {\dot{\gamma }}_{k+1}^{o}-{\dot{\gamma }}_{k}^{o} \end{array}\right]$$

The sensor measurement equation is:

(91) $$z_{k}=h_{k}\left(x_{k}\right)+v_{k}$$

where:

(92) $$h_{k}\left(x_{k}\right)=\text{arctan}\frac{\chi_{k}}{\gamma_{k}}$$

The measurement noise *v_k_* is a zero-mean white with covariance $R_{k}=\sigma_{z}^{2}={\left(1{}^{\circ}\right)}^{2}$. The Jacobian of *h_k_*(**x***_k_*) is calculated as:

(93) $$H_{k}=\left[\begin{array}{cccc} \frac{\gamma_{k}}{\chi_{k}^{2}+\gamma_{k}^{2}} & 0 & -\frac{\chi_{k}}{\chi_{k}^{2}+\gamma_{k}^{2}} & 0 \end{array}\right].$$

The initial FIM is $J_{0,1}^{-1}=\mathrm{diag}\left(c_{r}^{2},c_{v}^{2},c_{r}^{2},c_{v}^{2}\right)$, where the standard deviation of position is *c_r_* and standard deviation of velocity is *c_v_*.

The errors in cardinality mismatches are:

(94) $$\begin{array}{l} e_{1}={\left[\begin{array}{cccc} c_{r} & c_{v} & c_{r} & c_{v} \end{array}\right]}^{T},\\ e_{0}={\left[\begin{array}{cccc} c_{r} & c_{v} & c_{r} & c_{v} \end{array}\right]}^{T} \end{array}$$

Ownship is moving as a uniform circular motion. The dynamic equation of the observer is given by:

(95) $$x_{k+1}^{o}=\Theta_{k}x_{k}^{o}$$

(96) $$\Theta_{k}=\left[\begin{array}{cccc} 1 & \text{sin}\left(\omega \text{T}\right)/\omega & 0 & \left(-1+\text{cos}\left(\left(\omega \text{T}\right)\right)/\omega \right)\\ 0 & \text{cos}\left(\omega \text{T}\right) & 0 & -\text{sin}\left(\omega \text{T}\right)\\ 0 & \left(1-\text{cos}\left(\omega \text{T}\right)\right)/\omega & 1 & \text{sin}\left(\omega \text{T}\right)/\omega \\ 0 & \text{sin}\left(\omega \text{T}\right) & 0 & \text{cos}\left(\omega \text{T}\right) \end{array}\right]$$

**Figure 3.**
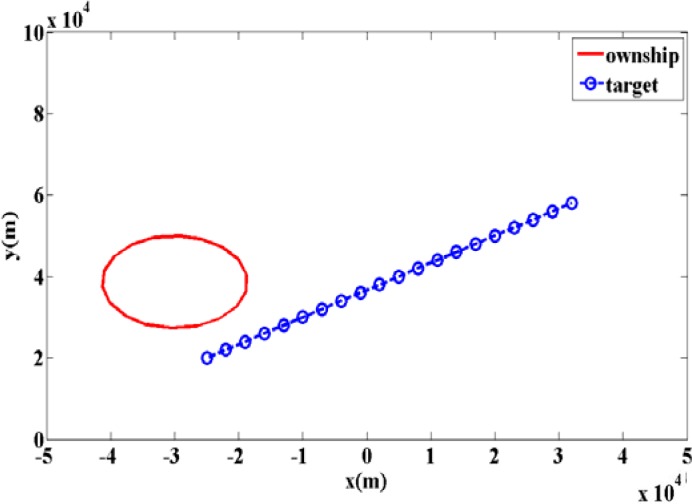
Bearing-only tracking scenario.

The initial target state vector $x_{1}^{t}={\left[\begin{array}{ccc} -25\;\text{km}\;150\;\text{m}/\text{s} & 20\;\text{km} & 100\;\text{m}/\text{s} \end{array}\right]}^{T}$ and the initial observer state vector $x_{1}^{o}={\left[\begin{array}{ccc} -30\;\text{km}\;200\;\text{m}/\text{s} & 50\;\text{km} & 0\;\text{m}/\text{s} \end{array}\right]}^{T}$. The time interval is *T* = 20 s. The initial target state standard variance is *c_r_* = 10,000 m *c_v_* = 100 m/s. The angular velocity is *ω* = 1.0125°/s. The target and observer trajectories are shown in Figure 3.

**Figure 4.**
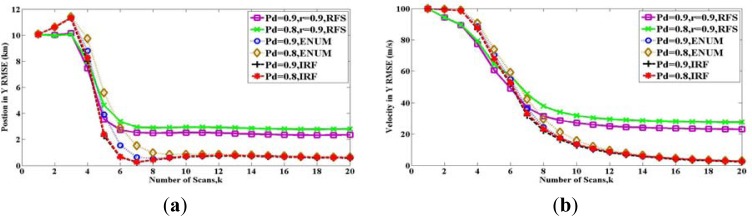
Comparisons of bounds with different detection probability P_D_: (**a**) Position in Y axes (**b**) Velocity in Y axes.

**Figure 5.**
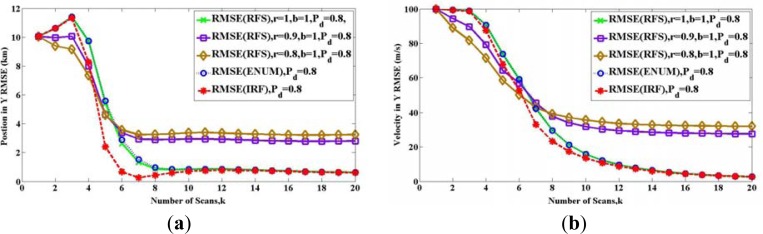
Comparisons of bounds with different maintenance probability r: (**a**) Position in Y axes (**b**) Velocity in Y axes.

**Figure 6.**
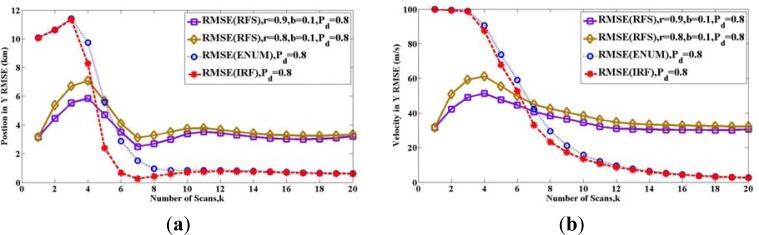
Comparisons of bounds with different initial existence probability b: (**a**) Position in Y axes (**b**) Velocity in Y axes.

Figures 4, 5 and 6 show the root-mean-square error (RMSE) bound between two sets *X_k_* and *X̂_k_*(*Z*_1_ ⋯ *Z_k_*) in (a) y-position and (b) y-velocity for twelve scans. Similar results can be also obtained for the x-axis of position and velocity.

In Figure 4, the two solid lines show the bound **P***_k_*(*RFS*) with different detection probability *P_D_*, for the case where the maintenance probability is *r* = 0.9 and the initial probability is *b* = 1. The meanings of these two parameters are indicated in (25)–(27). The two long dashed lines represent the PCRLBs calculated by IRF method **P***_k_*(*IRF*). The two short dashed lines represent the PCRLBs calculated by Enumeration method **P***_k_*(*ENUM*).

There are many lines in Figure 4, and they are compared in two ways. On the one hand, we can compare the two lines of **P***_k_*(*RFS*) with various detection probability *P_D_*. It shows that the bounds are increased, when the detection probability *P_D_* is reduced. Similar results can be seen both for **P***_k_*(*IRF*) and **P***_k_*(*ENUM*). Therefore, for the same method, the bigger detection probability *P_D_* is, the lower estimation bound is. On the other hand, comparing to PCRLB, the RFS bound **P***_k_*(*RFS*) is larger than **P***_k_*(*IRF*) and **P***_k_*(*ENUM*) after eight scans. The reason is that maintenance probability is *r* = 0.9, which introduces the uncertainty of target existence.

In Figures 5 and 6, the two solid lines show the RFS bound **P***_k_*(*RFS*) with different maintenance probability is *r* = 0.9 and *r* = 0.8. The red long dashed line represents the IRF PCRLB **P***_k_*(*IRF*). The blue short dashed line represents the ENUM PCRLB **P***_k_*(*ENUM*). In Figure 5, the initial probability remains *b* = 1. In Figure 6, the initial probability is *b* = 0.1, and thus there are two different models for calculating the RFS bounds **P***_k_*(*RFS*) in Figures 5 and 6. In Figure 5, the initial probability is *b* = 1 and maintenance probability remains *r* < 1, it means the target appears at the first scan and disappears with probability 1 − *r* in the subsequent scans. In Figure 6, the initial probability is *b* = 0.1 and maintenance probability is *r* < 1, it means new target appears at the first scan with probability 0.1 and enters in with probability 1 − *r* in the subsequent scans.

To the RFS bound **P***_k_*(*RFS*), the influence of parameter maintenance probability *r* is hard to indicate. In Figure 5, the initial probability remains *b* = 1 (same parameter as in Figure 2), and then we get something similar to Figure 2, where RFS bounds **P***_k_*(*RFS*) with *r* = 0.9 and *r* = 0.8 intersect PCRLB **P***_k_*(*ENUM*). But, there are still some differences in these two cases. In Figure 2, the bound **P***_k_*(*RFS*) with *r* = 0.9 is a little more than which with *r* = 0.8, while in Figure 5, the situation is similar at first but is reversed with time. In Figure 6, the initial probability is *b* = 0.1, RFS bound **P***_k_*(*RFS*) with *r* = 0.9 is always a little less than which with *r* = 0.8. By comparing Figures 2, 5 and 6, it is seen that there is no clear relationship between the value of the maintenance probability *r* and the value of RFS bound, but we can see that the RFS bounds **P***_k_*(*RFS*) with both *r* = 0.9 and *r* = 0.8 are more than PCRLB **P***_k_*(*ENUM*) and **P***_k_*(*IRF*).

The influence of the initial probability *b* is obvious. Comparing Figures 5 and 6, it is clear that at different scans, the initial probability *b* gives different contributions to the bound. As discussed in Section 4.2, the uncertainty of target existence and the method of calculating estimation error have the opposite effect on the RFS bound **P***_k_*(*RFS*). In Figure 6, at first, both the state and the estimation of target are empty with high probability, since the initial probability remains *b =* 0.1. Thus the error is zero as defined in (24). We can see that the RFS bounds **P***_k_*(*RFS*) in Figure 6 are significantly low in the initial scans. But after seven scans, the uncertainty of target existence contributes more to RFS bounds **P***_k_*(*RFS*). At last, the RFS bounds **P***_k_*(*RFS*) in Figure 6 are more than PCRLB **P***_k_*(*ENUM*) and **P***_k_*(*IRF*).

Admittedly, it is impossible to prove all the intersections of the lines in Figures 5 and 6, but it is clear that **P***_k_*(*RFS*) is more than **P***_k_*(*ENUM*) after seven scans. These results are similar to the result of the first example in Section 5.1, which is shown in the Figure 2. It is easy to conclude that allowing the disappearance of existing targets and the appearance of new targets, RFS bound **P***_k_*(*RFS*) is more than PCRLBs **P***_k_*(*IRF*)and **P***_k_*(*ENUM*) with time, by introducing the uncertainty of target existence.

**Figure 7.**
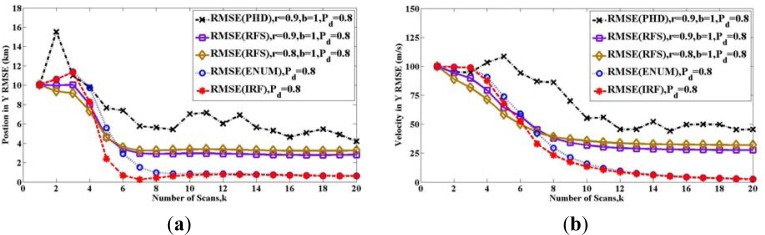
Comparisons of the performance of PHD filter and the bounds with different maintenance probability r: (**a**) Position in Y axes (**b**) Velocity in Y axes.

In Figure 7, we compare all the bounds with the performance obtained from a tracking algorithm in Figure 7. Following [22], we simulate a sequential Monte Carlo implementation of the PHD filter. For the prediction operator, at the first step, all particles are sampled with uniform intensity. Then, the state of the particles transform by (88). The particles are sampled for new target with a probability of 1 − *r* and uniform intensity. For the update operator, the likelihood function is a Gaussian intensity with the variance $R=\sigma_{r}^{2}$. The density of clutter is zero (*P_FA_* = 0). The RMSE is obtained by repeated (100 time) simulations.

As in Figure 7, it is clear that for the case where *r* = 0.9, *b* = 1 and *P_D_* = 0.8, the RMSE of this PHD filter cannot reach the RFS bound. The reason is that PHD is a first moment of the target posterior probability, and PHD filter is a tractable alternative to the optimal filter, but the RFS bound is deduced by the target posterior probability directly. Therefore, RFS bound can be reached only by the optimal filter in the framework of RFS, which is so computationally challenging that it must usually be approximated as denoted in [15].

In conclusion, allowing the disappearance of existing targets and the appearance of new targets, RFS bound **P***_k_*(*RFS*) is tighter than PCRLBs **P***_k_*(*IRF*) and **P***_k_*(*ENUM*) with time, by introducing the uncertainty of target existence. The initial target existing probability *b* can only influence at the initial some steps, but the maintenance probability *r* plays a leading role with time. Besides, the computational complexity of ENUM PCRLB grows exponentially with time. Therefore, when we consider reducing the amount of computation, IRF PCRLB is also an alternative.

## 6. Conclusions

The paper is devoted to comparison of the recently reported bounds within the framework of finite set statistics and the traditional PCRLB bounds, which are applicable to the case where the detection probability *P_D_* < 1 and the false alarm probability *P_FA_* = 0. This problem is firstly discussed when the target exists from the beginning to the end. When the target exists from the first scan to the last one, by the proof of two propositions, the RFS bound is similar with the ENUM PCRLB with four conditions and is always tighter than the IRF PCRLB. Then this problem is discussed when the target may appear or disappear. When the target appears and disappears with certain probability, these three bounds are compared by ballistic object tracking and bearings-only tracking applications. The simulation studies confirm the theoretical results and moreover, show that the RFS bound is tighter than the ENUM PCRLB and IRF PCRLB with time. It means that, the estimation error cannot reach the PCRLBs, but just reaches the RFS bound, when the target appears or disappears with certain probability. Additionally, the computational complexity of RFS bound grows exponentially with time. As ENUM PCRLB, the RFS bound is computationally feasible for a short time prediction, while IRF PCRLB can be applied to a long time prediction.
